# The imprinted *Phlda2* gene modulates a major endocrine compartment of the placenta to regulate placental demands for maternal resources

**DOI:** 10.1016/j.ydbio.2015.10.015

**Published:** 2016-01-01

**Authors:** S.J. Tunster, H.D.J. Creeth, R.M. John

**Affiliations:** Cardiff School of Biosciences, Cardiff University, Cardiff, Wales CF10 3AX, UK

**Keywords:** *Phlda2*, Placenta, Hormones, Epigenetics, Imprinting

## Abstract

Imprinted genes, which are expressed from a single parental allele in response to epigenetic marks first established in the germline, function in a myriad of processes to regulate mammalian development. Recent work suggests that imprinted genes may regulate the signalling function of the placenta by modulating the size of the endocrine compartment. Here we provide *in vivo* evidence that this hypothesis is well founded. Elevated expression of the imprinted *Pleckstrin homology-like domain, family a, member 2* (*Phlda2*) gene drives a reduction of the spongiotrophoblast endocrine compartment, diminished placental glycogen and asymmetric foetal growth restriction. Using both loss-of-function and gain-in-expression mouse models, here we further show that *Phlda2* exclusively modulates the spongiotrophoblast compartment of the placenta without significantly altering the composition of the trophoblast giant cell endocrine lineages that share a common progenitor with this lineage. Additionally, we show that *Phlda2* loss-of-function placentae contain nearly three times more placental glycogen than non-transgenic placentae. Remarkably, relative to a fully wild type scenario, wild type placentae also accumulate excessive glycogen. While loss-of-function of *Phlda2* increased both placental weight and placental glycogen, the weight of both mutant and non-transgenic fetuses was lower than that found in a fully wild type scenario indicating that excessive glycogen accumulation comes at the cost of foetal growth. This work firstly highlights a novel signalling function for the spongiotrophoblast in stimulating the global accumulation of placental glycogen. Furthermore, this work suggests that *Phlda2* manipulates the placenta's demands for maternal resources, a process that must be tightly regulated by epigenetic marks to ensure optimal foetal growth.

## Highlights

• *Phlda2* specifically restrains development of the spongiotrophoblast.
• The spongiotrophoblast is a major endocrine compartment of the placenta.
• The spongiotrophoblast locally and globally boosts glycogen accumulation.
• Excessive glycogen accumulation is associated with foetal growth restriction.
• *Phlda2* regulates placental demands from maternal resources.

## 1 Introduction

Genomic imprinting is an epigenetic phenomenon which drives the preferential expression of certain genes from one parental allele (John and Surani, 1996). The finding that some paternally silenced genes restrain foetal growth while some maternally silenced genes are growth promoting led to the suggestion that imprinting may have evolved in response to the different energetic contributions that male and female mammals make to their offspring (Moore and Haig, 1991) and the effectively antagonistic selective pressure acting on the mother–offspring relationship (Moore, 2011). However, another conflict exists in mammals because maternal resources are utilised by both the growing foetus and the developing extra-embryonic tissues. In some mammals these supporting tissues can consume more than half the maternal resources allocated to a pregnancy (Fowden, 1997, Carter, 2000) introducing the potential for competition between their energetic requirements and that of the growing foetus.

In mice, a significant number of imprinted genes functionally converge to regulate placental growth and development (Tunster et al., 2013). The mature mouse placenta supports foetal growth from approximately embryonic day (E) 9.5 until birth and is organised into the histologically distinct labyrinth zone, junctional zone and maternal decidua interspersed with trophoblast giant cells (TGCs) lining the maternal tissues and vasculature at specific sites (Rai and Cross, 2014). While the function of the labyrinth in placental transport is well established, the function of the spongiotrophoblast and the glycogen cell lineages, which reside within the junctional zone, has yet to be fully determined experimentally. Placental glycogen, which accumulates in the glycogen cells of the junctional zone from mid-gestation, may provide a store of easily mobilisable energy late in gestation to support the final stages of foetal growth (Coan et al., 2006) supported by numerous mouse models in which limited glycogen stores are associated with foetal growth restriction (Lefebvre, 2012). The function of the spongiotrophoblast lineage is less well understood but several placental lactogens (Prls) and pregnancy-specific glycoproteins (Psgs) are expressed from this lineage some of which have been shown to act on the mother to induce the physiological changes required for a successful pregnancy (Samaan et al., 1968, Bhattacharyya et al., 2002, Muller et al., 1999, Kammerer and Zimmermann, 2010, Wu et al., 2008). While this lineage is required for foetal viability (Guillemot et al., 1994, Ono et al., 2006), fetuses can survive to term with very little spongiotrophoblast (Oh-McGinnis et al., 2011). A reduced spongiotrophoblast has been linked to foetal growth restriction in several studies (Oh-McGinnis et al., 2011, Hitz et al., 2005, Zheng-Fischhofer et al., 2007, Withington et al., 2006, Tunster et al., 2010) while an expanded spongiotrophoblast may drive delayed parturition associated with foetal and maternal death (Denda et al., 2011). Loss of function of several maternally expressed imprinted genes results in an expansion of the spongiotrophoblast lineage (John and Hemberger, 2012), most recently *Sirh7*/*Ldoc1* (Naruse et al., 2014), suggesting that the paternal genome has selectively silenced genes that limit spongiotrophoblast-specific functions. However, while there are a number of mouse mutants in which alterations in the spongiotrophoblast lineage have been reported, these defects commonly occur alongside alterations in additional placental lineages. In particular, the glycogen cell lineage and four of the six distinct TGC lineages which share a common progenitor with the spongiotrophoblast (Rai and Cross, 2014, Hu and Cross, 2010, Simmons et al., 2007, Gasperowicz et al., 2013) confounding their functional assessment.

*Pleckstrin homology-like domain family A member 2 (Phlda2)* is a maternally expressed gene that maps to the imprinted domain on mouse distal chromosome 7, which encodes a PH domain-only protein (Frank et al., 1999, Qian et al., 1997). Prior to the formation of the mature mouse placenta, *Phlda2* is expressed most strongly in the ectoplacental cone and the visceral endoderm of the yolk sac (Frank et al., 1999, Dunwoodie and Beddington, 2002, Takao et al., 2012). The ectoplacental cone contains the *trophoblast specific protein alpha* (*Tpbpa*)-positive progenitors that give rise to the spongiotrophoblast, the glycogen cells and four of the six TGC subtypes of the mature placenta (Rai and Cross, 2014, Simmons et al., 2007, Gasperowicz et al., 2013, Simmons et al., 2008). Loss-of-function of *Phlda2* results in an enlarged placenta with an expanded junctional zone and more placental glycogen but without foetal overgrowth (Frank et al., 2002). Elevated expression, at two-fold the endogenous level, results in placental stunting, a loss of the spongiotrophoblast lineage and reduced placental glycogen accumulation but without an alteration in the representation of the glycogen cell lineage or the parietal trophoblast giant cells which line the maternal decidua (Tunster et al., 2010, Salas et al., 2004). Additionally, elevated *Phlda2* drives a late, asymmetric foetal growth restriction (Tunster et al., 2014). Taken together, these data suggested that *Phlda2* acts indirectly to restrain foetal growth by limiting the expansion of the spongiotrophoblast lineage, which is required to stimulate glycogen accumulation. However, the effects of elevated *Phlda2* gene dosage on all the TGCs lineages has not been reported. Moreover, a characterisation of the placental lineages in the context of loss-of-function has not been performed.

To further investigate the role of *Phlda2* in regulating the placental endocrine lineages, glycogen accumulation and foetal growth, we performed an examination of the placental lineages in the different *Phlda2* gene dosage mouse models and biochemically quantified placental glycogen at different stages of development in response to loss-of-function of *Phlda2*. Remarkably, in addition to the anticipated over accumulation of placental glycogen in response to loss-of-function of *Phlda2*, we noted a similar phenotypes in non-transgenic placenta sharing the uterine environment. Rather than supporting enhanced foetal growth, these stores came to the detriment of foetal growth identifying a novel role for *Phlda2* in regulating maternal resource allocation between the placenta and the foetus.

## 2 Materials and methods

### 2.1 Mouse strains and genotyping

Animal studies and breeding were approved by the Universities of Cardiff Ethical Committee and performed under a UK Home Office project license (RMJ). All mice were housed under standard conditions throughout the study on a 12 h light–dark cycle with lights coming on at 06.00 h with a temperature range of 21 °C±2 with free access to water (tap water) and standard chow. The *Phlda2* targeted allele (Frank et al., 2002) was crossed into the 129S2/SvHsd (Harlan, 129) strain background for +8 generations. The single copy *Phlda2* transgenic line *Phlda2*^*+/+*BACx1^ (Tunster et al., 2014) was maintained on the 129 background by paternal transmission. *Phlda2* deficient fetuses were generated by crossing *Phlda2*^*+/−*^ females with wild type males. *Phlda2*^*+/−*^ females were crossed with *Phlda2*^*+/+*BACx1^ males to generate four genotypes: *Phlda2*^*+/+*^ (non-transgenic; 1X), *Phlda2*^*−/+*^ (maternal KO; 0X), *Phlda2*^*+/+*BACx1^ (single copy *Phlda2* transgene; 2X) and *Phlda2*^*−/+*BACx1^ (single copy transgene plus maternal KO; 1X). 129 wild type colonies were maintained alongside the transgenic colonies. For recipient transfer experiments, 2-cell embryos were surgically transferred into E0.5 wild type 129 recipients mated with vasectomised males.

### 2.2 Quantitative RNA analysis

RNA was extracted from whole placenta following careful removal of membranes and umbilicus. Quantitative PCR of reverse transcribed RNA (RT-qPCR) was performed and analysed as described (Tunster et al., 2010, Schmittgen and Livak, 2008). RNA was hybridised to Affymetrix Mouse Gene 2.0 ST chips. Data was analysed essentially as described (Zhang et al., 2009) and using Partek Genomic suite. Genes significantly up in *Phlda2*^*−/+*^^)^ (0X) and significantly down in *Phlda2*^*+/+*BACx1^ (2X) E16.5 whole placenta were tested for enrichment of gene ontology molecular function and biological process using the Database for Annotation, Visualisation and Integrated Discovery (DAVID). Microarray data available in GEO repository accession *******.

### 2.3 *In situ* hybridisation and histological analyses

Placentas were fixed overnight in phosphate-buffered 4% paraformaldehyde, paraffin-embedded and 6 μm sections taken through the midline. Riboprobe preparation and *in situ* hybridisation were performed as previously described (Tunster et al., 2010, Tunster et al., 2012).

### 2.4 Weighing studies and biochemical determination of placental glycogen concentration

Foetal and placental wet weights were taken at the stated time points after a discernable plug and normalised. Genotyping data was obtained from yolk sac DNA as previously described (Frank et al., 2002, John et al., 2001). Glycogen was extracted from whole placenta, and resuspended in 1 ml of H_2_O and assayed according to the method of Lo et al. (1970) at a dilution of 1 in 2.

### 2.5 Statistical analyses

Statistical significance (probability values) was determined using the Student's *t*-Test (two tailed distribution and two sample unequal variance) for comparisons between knockout and control littermates. Comparisons with the fully wild type scenario were undertaken using ANOVA with Bonferroni correction.

## 3 Results

### 3.1 *Phlda2* supresses the expansion of the spongiotrophoblast without altering the representation of the TGC lineages

**Fig. 1:**
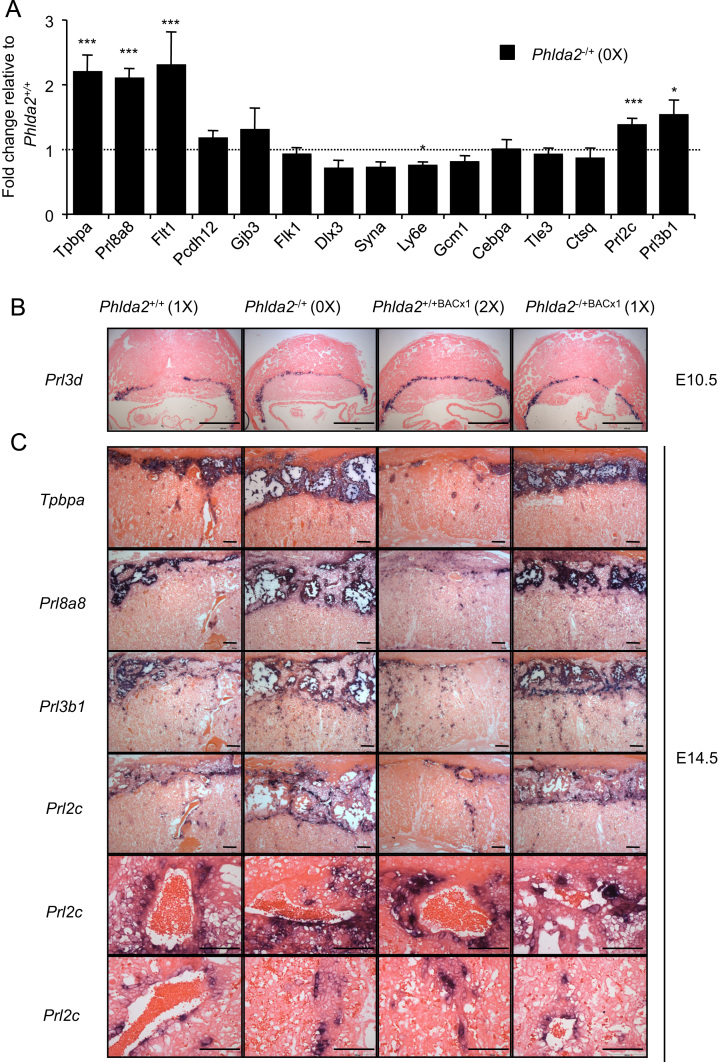
*Phlda2* restrains the expansion of the spongiotrophoblast lineage without significantly altering other trophoblast cell lineages. A. RT-qPCR analysis of placental lineage markers mRNA levels between E14.5 *Phlda2*^*+/+*^(non-transgenic; 1X) and *Phlda2*^*−/+*^(0X) placentae from littermates examined on the 129 genetic background. B. *In situ* hybridisation with *Prl3d (Pl1)* of midline sections from E10.5 *Phlda2*^*+/+*^(1X), *Phlda2*^−^^*/+*^(0X), *Phlda2*^*−/+BACx1*^(2X) and *Phlda2*^*−/+BACx1*^ (1X) 129 placenta. C. *In situ* hybridisation of midline sections E14.5 placenta with *Tpbpa*, *Prl8a8, Prl3b1* (*Pl2)* and *Prl2c (Plf)* (scale bar=200 μm). *Prl2c* also shown at a higher magnification illustrating similar staining of the canal and the spiral artery TGC (scale bar=100 μm). For the RT-qPCR analysis, *N*=4 placenta per genotype (2 *vs* 2 from 2 independent litters); error bars represent SEM. Statistical significance calculated using *t*-test. ^NS^*P*>0.05, *P<0.05, **P<0.01, and ***P<0.005.

A characterisation of placental lineages in response to loss of *Phlda2* function was undertaken after breeding the original line carrying a targeted deletion of *Phlda2* onto a 129S2/SvHsd (129) genetic background. *Phlda2*^*−/+*^ (maternal KO; 0X) placenta expressed *Tpbpa*, a gene expressed by both the spongiotrophoblast and the glycogen cell lineage (Lescisin et al., 1988), at 2.20-fold (±0.26; *p*=3.77×10^−4^) the normal level (Fig. 1A, and Table S1). *Prl8a8*, a gene expressed exclusively in spongiotrophoblast cells (Simmons et al., 2008), was elevated by 2.10-fold (±0.15; *p*=8.78±10^−5^) and *FMS-like tyrosine kinase 1* (*Flt1),* a marker of the junctional zone (Breier et al., 1995*)* was elevated by 2.31-fold (±0.51; *p*=0.00551). Expression of glycogen cell markers (*Gjb3*/*Cx31* and *Pcdh12*) and traditional markers of the labyrinth was comparable to wild type (Fig. 1A). These data were reciprocal to our findings in the gain-in-expression *Phlda2* model (Tunster et al., 2010, Tunster et al., 2014) confirming the inverse relationship between *Phlda2* gene dosage and the spongiotrophoblast but not the glycogen cell lineage. Some markers of the TGC lineages, *Tle3* and *Ctsq*, were expressed normally whereas *Prl2c (Plf)* and *Prl3b1,* which are expressed in both the spongiotrophoblast and TGCs, were elevated in response to loss-of-function of *Phlda2* (Fig. 1A) suggesting that some TGC lineages may also be altered in this model. To further refine the analysis, four placental genotypes were generated from crosses between *Phlda2*^*+/*^^−^ females and males carrying a single copy of a transgene spanning the *Phlda2* locus: *Phlda2*^*+/+*^ (non-transgenic; 1X), *Phlda2*^*−/+*^ (maternal KO; 0X), *Phlda2*^*+/+*BACx1^ (single copy *Phlda2* transgene; 2X) and *Phlda2*^*−/+*BACx1^ (single copy transgene plus maternal KO; 1X). *In situ* hybridisation performed with *Prl3d*, an exclusive marker of the parietal TGC lineage at E10.5 (Simmons et al., 2008), excluded any gross alterations in this lineage (Fig. 1B). Hybridisation with *Tpbpa* and *Prl8a8* at E14.5 further highlighted the inverse relationship between *Phlda2* gene dosage and the spongiotrophoblast with an expansion of this lineages occurring in response to the decreasing expression of *Phlda2*. Hybridisation with *Prl2b1* and *Prl2c*, which identify the canal, sinusoidal and spiral artery TGCs (Simmons et al., 2007), was similar irrespective of *Phlda2* gene dosage (Fig. 1C) indicating that these lineages were relatively unaffected by the loss-of-function of *Phlda2*. These data identified *Phlda2* as a gene that acts exclusively to constrain the expansion of the spongiotrophoblast lineage without significantly altering the cellular composition of other trophoblast lineages which share a common progenitor.

### 3.2 *Phlda2* regulates the expression of several placental hormones

**Fig. 2:**
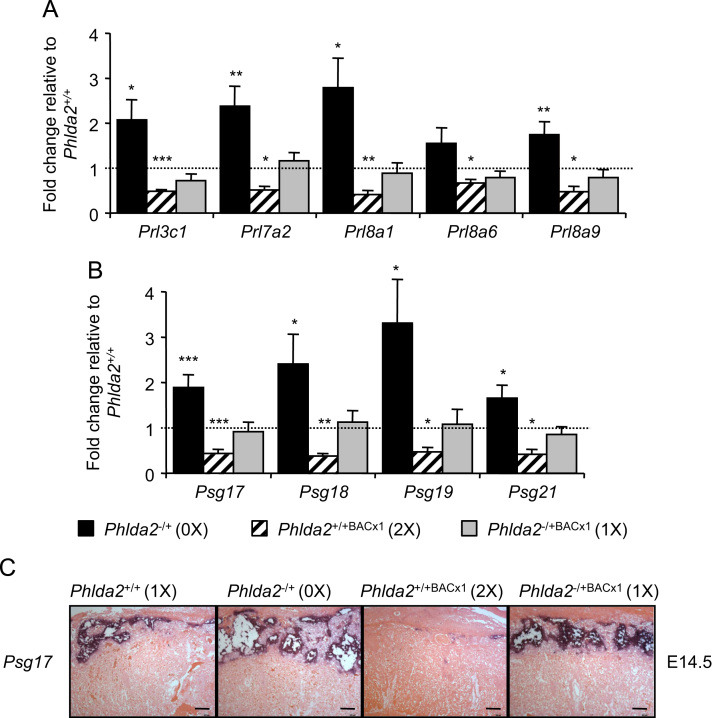
*Phlda2* indirectly suppresses the expression of key placental hormones. A. RT-qPCR comparison of *Prls* at E14.5 between *Phlda2*^*+/+*^(1X), *Phlda2*^*−/+*^(0X), *Phlda2*^*−/+BACx1*^(2X) and *Phlda2*^*−/+BACx1*^(1X) 129 placentae. B. RT-qPCR comparison of *Psgs* at E14.5. C. *In situ* hybridisation of midline sections E14.5 placentae with *Psg17* (scale bar=200 μm). For the RT-qPCR analysis, *N*=4 placenta per genotype (2 *vs* 2 from 2 independent litters); error bars represent SEM. Statistical significance calculated using *t*-test. ^NS^*P*>0.05, *P<0.05, **P<0.01, and ***P<0.005.

The spongiotrophoblast expresses a number of *Prls* (Simmons et al., 2008)*,* genes that encode placental lactogens that act on the mother during pregnancy to induce changes required for a successful pregnancy (John, 2013). Several *Prl* members were elevated in the *Phlda2*^*−/+*^ (maternal KO; 0X) placenta and reduced in the *Phlda2*^*+/+*BACx1^ (2X) placenta (Fig. 2A**,**Table S1). The spongiotrophoblast is also a major source of pregnancy specific glycoproteins (*Psgs*), another large gene family important for pregnancy (McLellan et al., 2005). RT-qPCR analysis of the most abundantly expressed *Psg*s during mid gestation, *Psg17*, *Psg18*, *Psg19* and *Psg21* (Fig. 2B), and *in situ* hybridisation with *Psg17* (Fig. 2C) revealed a reciprocal relationship between *Phlda2* expression and the *Psgs. In situ* hybridisation (Figs. 1B, C and 2C) and RT-qPCR analyses (Table S1) on double transgenic placenta carrying both the transgene and the maternally inherited targeted *Phlda2* allele (1X) formally assigned these changes to *Phlda2*.

**Fig. 3:**
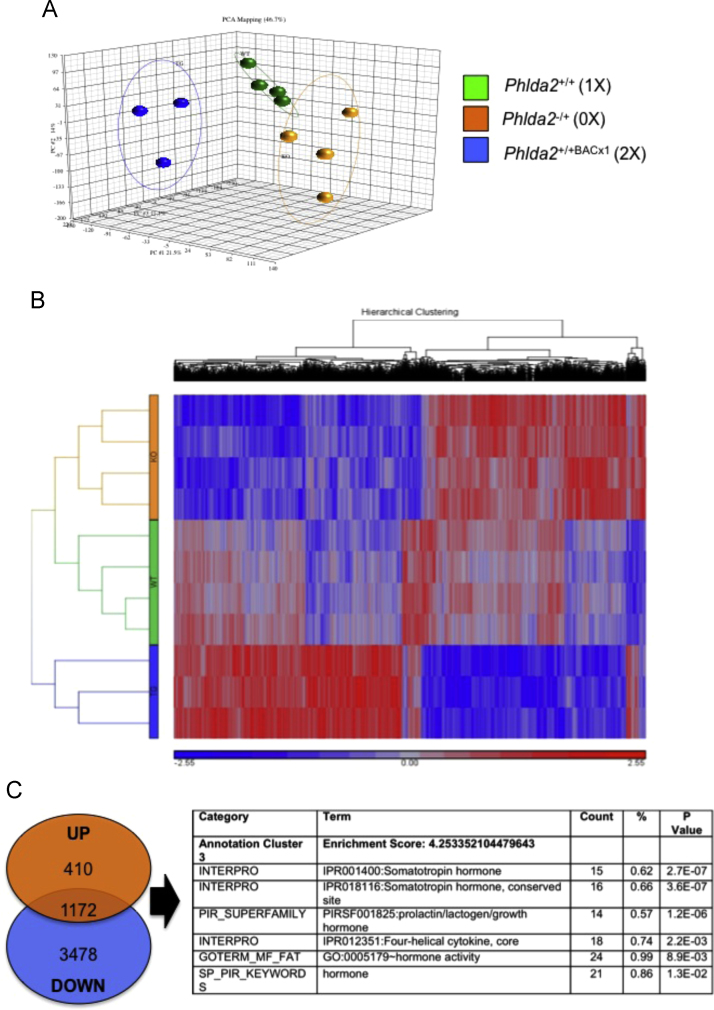
Microarray analysis identifies the transcriptomic signature of the spongiotrophoblast. A. Three-dimensional principle components analysis plot clustering placental gene expression with genotypes. B. Heat map showing genes significantly altered in relations to changes in *Phlda2* gene dosage. The colour represents the expression level of the gene. Red represents high expression, while green represents low expression. The expression levels are continuously mapped on the colour scale provided at the bottom of the figure. *Phlda2*^*−/+*^(0X) and *Phlda2*^*+/+BACx1*^(2X) placenta show a reciprocal pattern of expression. C. Enrichment for placental hormones after pathways analysis of genes significantly UP in *Phlda2*^*−/+(129)*^(0X) and significantly DOWN in *Phlda2*^*+/+BACx1*^(2X) placenta (*p*≤0.05) presumed to originate within the spongiotrophoblast. Supplemental Table 1 list the DAVID results and the UP DOWN annotated probe sets with a significant differences in gene expression at *P*≥0.05.

A more objective analysis of gene expression changes was performed using an Affymetrix mouse microarray to profile gene expression on E16.5 *Phlda2*^*+/+*^ (1X), *Phlda2*^*−/+*^ (0X) and *Phlda2*^*+/+*BACx1^ (2X) placenta (*N*=3–4 of each genotype, two independent litters for each genotype). Data was initially analysed at the genome wide level using a Bioconductor package Limma (Linear Model for Microarray data Analysis) written in R statistical software to identify differentially expressed genes between all pair-wise comparisons of the groups (Fig. 3A and B). Database for Annotation, Visualisation and Integrated Discovery (DAVID) was used to generate molecular function and biological processes pathway data on the unselected dataset highlighting significant changes in genes involved in cell cycle, cytokine–cytokine interactions and sphingolipid metabolism (Table S2). Genes both significantly up in *Phlda2*^*−/+*^ placenta and significantly down in *Phlda2*^*+/+*BACx1^ E16.5 whole placenta likely represent the spongiotrophoblast transcriptome (Fig. 3C, and Table S3). A dataset of these UP DOWN genes was further analysed by DAVID giving a functional annotation clustering analysis output which highlighted cytoskeleton pathways, cell division pathways and the protein superfamily prolactin/lactogen/growth hormone cluster (Cluster 3 Enrichment Score: 4.25) (Tables S4 and S5). Within cluster 3 were a number of *placental prolactins* known to be expressed within the spongiotrophoblast (Simmons et al., 2008) and *secretin,* also highly expressed within the spongiotrophoblast (Knox et al., 2011) (Table S6). This data, together with our RT-qPCR and *in situ* analyses (Fig. 1, Fig. 2), confirmed *Phlda2* as a major rheostat for placental hormone gene expression.

### 3.3 Excessive accumulation of placental glycogen in *Phlda2*-deficient placenta

**Fig. 4:**
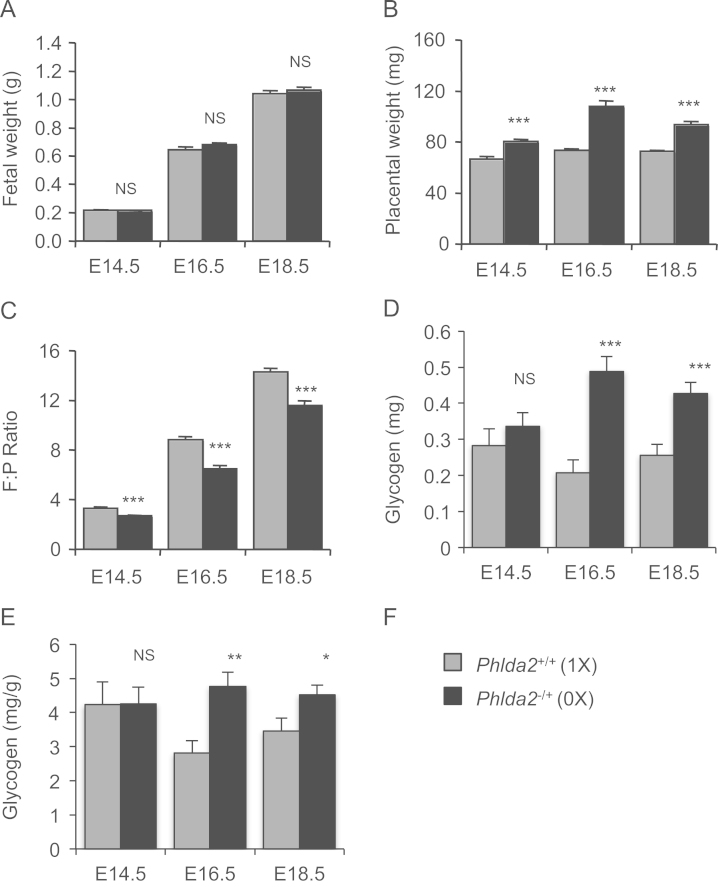
Influence of *Phlda2* deficiency on foetal and placental weights, and glycogen accumulation. A. Weights of non-transgenic and *Phlda2*^*−/+*^(0X) fetuses at the indicated gestational stages. *Phlda2*^*−/+*^(0X) fetuses weigh the same as their non-transgenic counterparts at each time point. B. Weights of placentae at the indicated gestational stages. *Phlda2*^*−/+*^(0X) placenta weigh more than their non-transgenic counterparts at each timepoint. C. Comparison of F:P ratios on the 129 background. D. Direct biochemical determination of total glycogen (mg) stored in *Phlda2*^*+/+*^ and *Phlda2*^*−/+*^(0X) placenta at E14.5, E16.5 and E18.5. *Phlda2*^*−/+*^(0X) placenta contain significantly more glycogen than non-transgenic placenta at E16.5 and E18.5. E. Milligrams (mg) of glycogen stored per gram of placenta in *Phlda2*^*+/+*^ and *Phlda2*^*−/+*^(0X) placenta at E16.5 and E18.5. Numbers of samples given in Supplemental Table S7. Error bars represent SEM. Statistical significance calculated using *t*-test: ^NS^*P*>0.05, *P<0.05, **P<0.01, and ***P<0.005.

*Phlda2*-deficiency results in a transient foetal growth restriction in C57BL/6 (BL6) mice (Frank et al., 2002, Salas et al., 2004). Given the less favourable foetal:placental ratio in 129 mice (Tunster et al., 2012), we asked whether foetal overgrowth might manifest on this genetic background. *Phlda2*^*−/+*^ fetuses were similar in weight to their 129 non-transgenic counterparts at E14.5, E16.5 and E18.5 (Fig. 4A). *Phlda2*^*−/+*^ placenta weighed more than non-transgenic at each time point with a maximal increase of 50% at E16.5 (*p*=7.42×10^−8^) (Fig. 4B). *Phlda2*-deficiency resulted in a lower F:P ratios (Fig. 4C) particularly evident at E16.5 with a change in ratio from 8.8±0.26 to 6.4±0.30 (73.1% of control; *p*=2.75×10^−6^).

*Phlda2-*deficiency resulted in increased Periodic Acid Schiff (PAS) staining for glycogen (Tunster et al., 2010, Frank et al., 2002). When we biochemically determined the amount of glycogen stored in the 129 placenta, *Phlda2-*deficiency resulted in greater stores of placental glycogen, as anticipated, with a maximal difference in total glycogen at E16.5 of 2.3-fold (129; Fig. 4D**)**. These differences were maintained when placental weights were taken into account (Fig. 4E).

### 3.4 Global foetal growth restriction

**Fig. 5:**
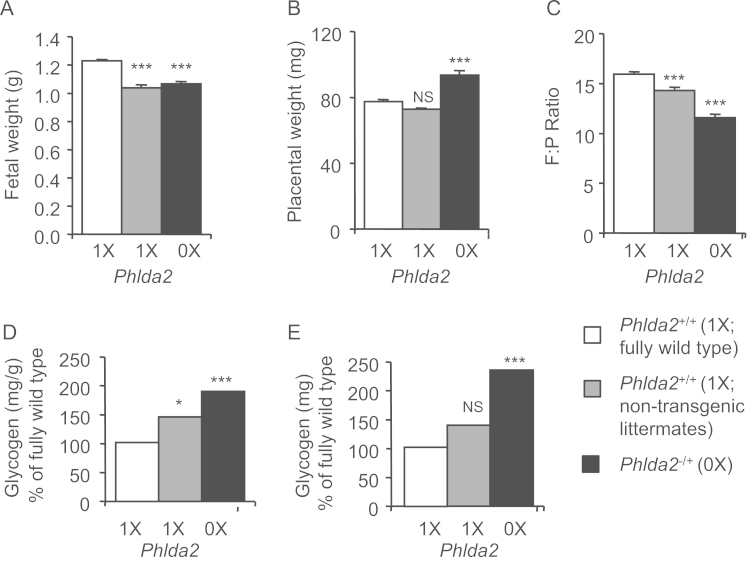
*Phlda2* deficiency drives global foetal growth restriction. A. Weights of non-transgenic and *Phlda2*^*−/+*^(0X) fetuses compared with true 129 wild type values (wild type fetuses carried by wild type females). Both genotypes weigh less than fully wild type fetuses. B. Weights of non-transgenic and *Phlda2*^*−/+*^(0X) placenta compared with true 129 wild type values. *Phlda2*^*−/+(129)*^(0X) placenta weigh considerably more than fully wild type placenta. C. Comparison of F:P ratios between non-transgenic, *Phlda2*^*−/+(129)*^(0X) and true 129 wild type. Both the non-transgenic and the *Phlda2*^*−/+*^ratios are significantly different to the fully wild type ratio. D. Direct biochemical determination of glycogen in *Phlda2*^*+/+*^ and *Phlda2*^*−/+*^(0X) placenta at E18.5 as a percentage of true 129 wild type values expressed as total amount (mg) and as a proportion of placental weight (mg/g placenta). *Phlda2*^*−/+*^(0X) placenta and the non-transgenic placenta both contain significantly more glycogen than fully wild type placenta on the 129 genetic background when placental weights are taken into account. Numbers of samples given in Supplemental Table S8. Error bars represent SEM. Statistical significance calculated using one way ANOVA with Bonferroni correction for multiple comparisons: ^NS^*P*>0.05, *P<0.05, **P<0.01, and ***P<0.005.

Given the function of *Phlda2* in restraining the spongiotrophoblast lineage and thus, indirectly, the production of placental hormones, loss-of-function of *Phlda2* might promote the accumulation of placental glycogen and potentially support the increased growth of both the loss-of-function fetuses and their non-transgenic counterparts sharing the intrauterine environment. The within litter comparison would not necessary exclude this possibility. To address this, data on *Phlda2-*deficient fetuses and their non-transgenic littermates were compared to those from fully wild type litters at E18.5 collected concurrently using the parent strain. Remarkably, rather than an anticipated increase in foetal weights, we observed a 15% reduction in weight of both *Phlda2*^*−/+*^ and *Phlda2*^*+/+*^ compared to fully wild type litters (Fig. 5A). Non-transgenic placentae were similar in weight to fully wild-type placentae (Fig. 5B). Consequently the F:P ratios for both the *Phlda2-*deficient scenario and the non-transgenic scenario were both significantly different to the fully wild-type scenario (Fig. 5C). Remarkably, while placental glycogen stores from non-transgenic placenta at E18.5 contained significantly less glycogen per gram of placenta than the *Phlda2*-deficient placenta, they contained more glycogen overall than the fully wild type placenta (Fig. 5D). Phenotypes in the non-transgenic samples sharing the intrauterine environment with the transgenic individuals highlighted a global effect on the entire litter as a result of localised loss-of-function of *Phlda2*.

**Fig. 6:**
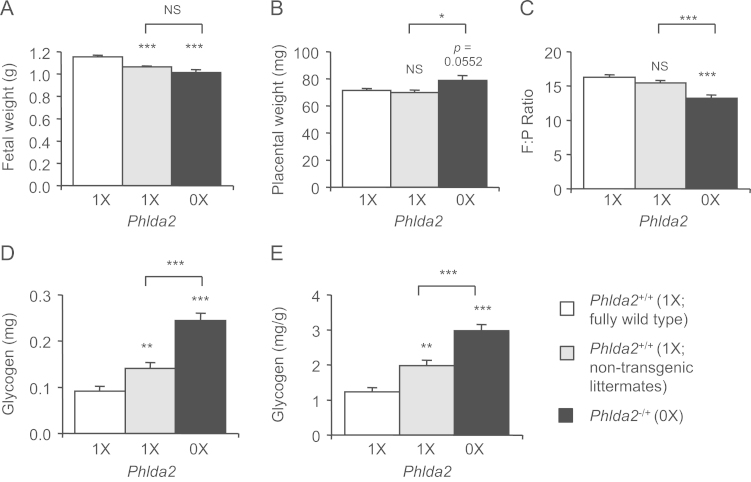
Exclusion of a maternal genotype phenotype. A. Comparison of wet weights of *Phlda2*^*−/+*^ (0X) and non-transgenic fetuses carried by wild type dams generated by recipient transfer compared with fully wild type foetal weights at E18.5. Both foetal genotypes weight less than fully wild type fetuses. B. Comparison of wet weights of *Phlda2*^−^^*/+*^ (0X) and non-transgenic placenta carried by wild type dams generated by recipient transfer compared with fully wild type placental weights at E18.5. C. F:P ratios from data in A and B. D. Comparison of total placental glycogen (mg) at E18.5. Both placental genotypes contain more glycogen than fully wild type placenta. E. Comparison of placental glycogen per gram of placenta (mg/g) at E18.5. Both placental genotypes contain more glycogen when placental weight is taken into account than fully wild type placenta. Numbers of samples given in Supplemental Table S9. Error bars represent SEM. Statistical significance calculated using one way ANOVA with Bonferroni correction for multiple comparisons: ^NS^*P*>0.05, *P<0.05, **P<0.01, and ***P<0.005.

The litters we examined were carried by dams inheriting the targeted *Phlda2* allele, albeit paternally and thus the inactive allele. While *Phlda2* is imprinted strongly in the placenta and extraembryonic membranes, functional imprinting is less well maintained in embryonic and adult tissues (Qian et al., 1997). To formally exclude a haploinsufficiency phenotype in the dams, *Phlda2*-deficient embryos and their non-transgenic controls were transferred into wild-type recipient mothers. The weights of both *Phlda2*^−/+^ (KO) and non-transgenic fetuses were significantly lighter when compared to fully wild-type litters (Fig. 6A). *Phlda2*^−/+^ (KO) placental weights were significantly increased (Fig. 6B) and the F:P ratio was lower (Fig. 6C). Importantly, whether examined as total glycogen or as glycogen per gram of placenta, the *Phlda2*^−/+^ (KO) and non-transgenic values were significantly higher than fully wild-type control values (Fig. 6D and E). These data excluded a maternal genotype-effect and identified a novel function for the spongiotrophoblast in promoting the global accumulation of placental glycogen.

## 4 Discussion

Here we show that the imprinted *Phlda2* gene acts in a lineage-specific manner to exclusively modulate the size of the spongiotrophoblast compartment of the mature mouse placenta. *Phlda2* is the first gene described which has this specific function allowing the *in vivo* assessment of the function of this lineage. Using both loss-of-function and gain-in-expression models we show that, via the spongiotrophoblast, *Phlda2* negatively regulates the expression of a number of key placental hormones. We show that an expanded spongiotrophoblast compartment, driven by loss-of-function of *Phlda2,* drives an inappropriate accumulation of placental glycogen both locally, in the genetically modified individuals, and also globally in individuals that were genetically wild type. This work identifies a novel signalling function for the spongiotrophoblast. The presence of foetal growth restriction in both the genetically modified individuals and their non-transgenic counterparts further suggests that *Phlda2* balances resource allocation between the foetus and the placenta, a process that must be precisely regulated for optimal foetal growth.

**Fig. 7:**
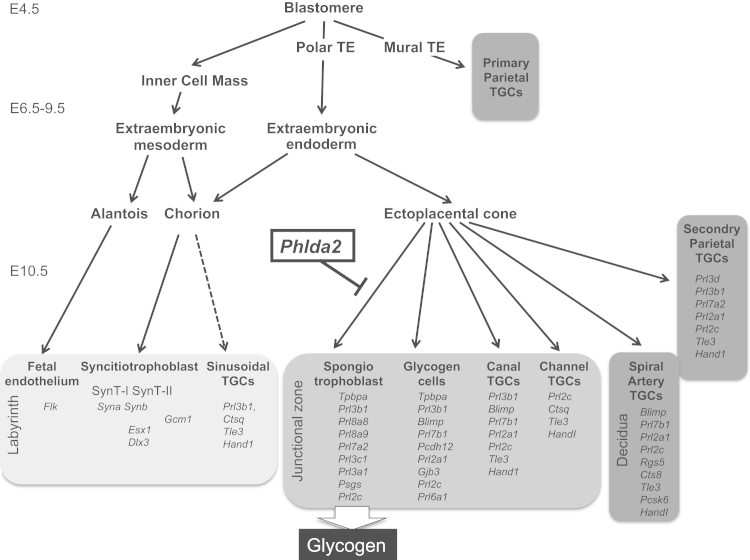
Summary of the cell autonomous and non-cell autonomous functions of *Phlda2* in regulating placental glycogen accumulation. Trophoblast lineage allocation and lineage markers adapted from Rai and Cross (2014).

*Phlda2* is the only gene to date which acts to limit the size of the spongiotrophoblast compartment of the mouse placenta without significantly altering the contribution of additional placental lineages that share a common progenitor (Rai and Cross, 2014). This suggests that *Phlda2* acts in placental development after these lineage decisions are made but before *Phlda2* expression subsides (Fig. 7). This unique specificity for the spongiotrophoblast allowed us to explore the function of the spongiotrophoblast. One key finding from this study was that an expanded spongiotrophoblast drove the excessive accumulation of placental glycogen both directly, within the loss-of-function placenta, and also indirectly in the non-transgenic placenta sharing the uterine environment. To our knowledge, this is the first example of a gene modification affecting the phenotype in wild type conceptuses of the same litter. However, very few studies make the comparison with fully wild type data. These data suggest that the spongiotrophoblast produces a signal that acts locally and at a distance to stimulate glycogen accumulation. The spongiotrophoblast expresses a number of placental prolactins (Simmons et al., 2008) and pregnancy specific glycoproteins (McLellan et al., 2005). Consequently, by changing the size of the spongiotrophoblast compartment, *Phlda2* indirectly and negatively regulates the expression of several placental hormones. Many of these placental hormones act on the maternal system to channel nutrients to the foetus and ensure a healthy and successful pregnancy (Samaan et al., 1968, Bhattacharyya et al., 2002, Muller et al., 1999, Kammerer and Zimmermann, 2010, Wu et al., 2008). One explanation for our findings is that an increased signal (*Phlda2* loss-of-function) leads to increased nutrient availability potentially explaining increased placental glycogen in both genotypes. However, we observed foetal growth restriction of both the *Phlda2* KO fetuses and their non-transgenic, genetically wild-type littermates. We could discount a haploinsufficiency phenotype in the dams, which carry the paternal targeted allele, because embryos transferred into wild-type recipients also displayed both foetal growth restriction and excessive placental glycogen of both genotypes (Fig. 6). Another explanation is that the defect lies at the level of foetal uptake of glucose or the ability to transport it across the placenta to the foetus such that placental glycogen accumulates because it is not utilised. A third explanation is that the spongiotrophoblast produces a signal demanding nutrients to support placental growth. When the signal is too high, as in the *Phlda2* loss-of-function model, it outcompetes the demand signal from the foetus resulting in the diversion of maternal resources away from supporting foetal growth and towards supporting placental growth. This latter explanation is plausible as the placenta is a highly metabolic organ consuming maternal energy in order to support the active transport of nutrients to the foetus and also the production and secretion of vast quantities of placental hormones (John, 2013). In mammals, more than half of the total uterine glucose and oxygen uptake is channelled to the uteroplacental tissues (Fowden, 1997, Carter, 2000). A larger placenta, observed in the *Phlda2* loss-of-function model, would require a greater proportion of maternal resources that might occur to the detriment of foetal growth. While further work is required to determine the precise mechanism driving foetal growth restriction in this model, this study has demonstrated that a precisely regulated dose of *Phlda2* is essential for optimal foetal growth with both loss-of-function and gain-in-expression driving foetal growth restriction in mice.

*Phlda2* does not appear to follow the straightforward rules applied to a substantial number of imprinted genes whereby paternally expressed genes promote placental and foetal growth whereas maternally expressed genes restrain placental and foetal growth. *Phlda2* resides within a complex imprinted domain spanning several maternally expressed genes including *Cdkn1c*, a potent embryonic growth restriction gene (Andrews et al., 2007, Tunster et al., 2011). Neither loss-of-function of *Phlda2* nor loss-of-function of *Cdkn1c* alone in mice results in the increase in birth weight predicted by the genomic conflict theory. It may be that loss of function of such critically important genes has too severe an impact on foetal development. Alternatively, the combined alteration may be required to generate larger offspring. Our data from mouse models would predict an increase in foetal weight, due to reduced *Cdkn1c* expression, alongside an increase in the signalling function of the placenta, due to reduced *Phlda2* expression, thus retaining the balance between foetal growth and placental demands on maternal resources. Co-imprinting of these two genes, which occurred after marsupials diverged for Eutherian mammals (Suzuki et al., 2005, Suzuki et al., 2011), may consequently have played an important role in the evolution of modern day mammals that give birth to relatively mature offspring.

In conclusion, we have shown that *Phlda2* modulates the signalling function of the placenta to limit the accumulation of placental glycogen and placental growth. While this does not provide a facile explanation for paternal silencing of *Phlda2*, our work highlights the complex relationship between the mother, the foetus and the placenta whose requirements must be carefully balanced for an optimal reproductive success.

## Competing interests statement

The authors declare that there is no conflict of interest financial or otherwise associated with this submission.

## Author contributions

SJT performed the bulk of the experiments, analysed data and contributed to the writing of the manuscript. HDJC performed the microarray analysis and some of the animal work. RMJ conceived and designed the experiment, interpreted the data and wrote the paper.

## Footnotes

Appendix A Supplementary data associated with this article can be found in the online version at doi:10.1016/j.ydbio.2015.10.015.

## References

[bib1] Andrews S.C. (2007). Cdkn1c (p57Kip2) is the major regulator of embryonic growth within its imprinted domain on mouse distal chromosome 7. BMC Dev. Biol..

[bib2] Bhattacharyya S., Lin J., Linzer D.I. (2002). Reactivation of a hematopoietic endocrine program of pregnancy contributes to recovery from thrombocytopenia. Mol. Endocrinol..

[bib3] Breier G., Clauss M., Risau W. (1995). Coordinate expression of vascular endothelial growth factor receptor-1 (flt-1) and its ligand suggests a paracrine regulation of murine vascular development. Dev. Dyn..

[bib4] Carter A.M. (2000). Placental oxygen consumption. Part I: in vivo studies – a review. Placenta.

[bib5] Coan P.M. (2006). Origin and characteristics of glycogen cells in the developing murine placenta. Dev. Dyn..

[bib6] Denda K. (2011). Nrk, an X-linked protein kinase in the germinal center kinase family, is required for placental development and fetoplacental induction of labor. J. Biol. Chem..

[bib7] Dunwoodie S.L., Beddington R.S. (2002). The expression of the imprinted gene Ipl is restricted to extra-embryonic tissues and embryonic lateral mesoderm during early mouse development. Int. J. Dev. Biol..

[bib8] Fowden A.L. (1997). Comparative aspects of fetal carbohydrate metabolism. Equine Vet. J..

[bib9] Frank D. (1999). A novel pleckstrin homology-related gene family defined by Ipl/Tssc3, TDAG51, and Tih1: tissue-specific expression, chromosomal location, and parental imprinting. Mamm. Genome.

[bib10] Frank D. (2002). Placental overgrowth in mice lacking the imprinted gene Ipl. Proc. Natl. Acad. Sci. USA.

[bib11] Gasperowicz M. (2013). The transcriptional co-repressor TLE3 regulates development of trophoblast giant cells lining maternal blood spaces in the mouse placenta. Dev. Biol..

[bib12] Guillemot F. (1994). Essential role of Mash-2 in extraembryonic development. Nature.

[bib13] Hitz C. (2005). Progressive loss of the spongiotrophoblast layer of Birc6/Bruce mutants results in embryonic lethality. Genesis.

[bib14] Hu D., Cross J.C. (2010). Development and function of trophoblast giant cells in the rodent placenta. Int. J. Dev. Biol..

[bib15] John R., Hemberger M. (2012). A placenta for life. Reprod. Biomed. Online.

[bib16] John R.M. (2001). Distant cis-elements regulate imprinted expression of the mouse p57( Kip2) (Cdkn1c) gene: implications for the human disorder, Beckwith–Wiedemann syndrome. Hum. Mol. Genet..

[bib17] John R.M. (2013). Epigenetic regulation of placental endocrine lineages and complications of pregnancy. Biochem. Soc. Trans..

[bib18] John R.M., Surani M.A. (1996). Imprinted genes and regulation of gene expression by epigenetic inheritance. Curr. Opin. Cell Biol..

[bib19] Kammerer R., Zimmermann W. (2010). Coevolution of activating and inhibitory receptors within mammalian carcinoembryonic antigen families. BMC Biol..

[bib20] Knox K. (2011). Global hormone profiling of murine placenta reveals Secretin expression. Placenta.

[bib21] Lefebvre L. (2012). The placental imprintome and imprinted gene function in the trophoblast glycogen cell lineage. Reprod. Biomed. Online.

[bib22] Lescisin K.R., Varmuza S., Rossant J. (1988). Isolation and characterization of a novel trophoblast-specific cDNA in the mouse. Genes Dev..

[bib23] Lo S., Russell J.C., Taylor A.W. (1970). Determination of glycogen in small tissue samples. J. Appl. Physiol..

[bib24] McLellan A.S. (2005). Structure and evolution of the mouse pregnancy-specific glycoprotein (Psg) gene locus. BMC Genom..

[bib25] Moore T. (2011). Review: parent–offspring conflict and the control of placental function. Placenta.

[bib26] Moore T., Haig D. (1991). Genomic imprinting in mammalian development: a parental tug-of-war. TIG.

[bib27] Muller H. (1999). Uterine natural killer cells are targets for a trophoblast cell-specific cytokine, prolactin-like protein A. Endocrinology.

[bib28] Naruse M. (2014). Sirh7/Ldoc1 knockout mice exhibit placental P4 overproduction and delayed parturition. Development.

[bib29] Oh-McGinnis R., Bogutz A.B., Lefebvre L. (2011). Partial loss of Ascl2 function affects all three layers of the mature placenta and causes intrauterine growth restriction. Dev. Biol..

[bib30] Ono R. (2006). Deletion of Peg10, an imprinted gene acquired from a retrotransposon, causes early embryonic lethality. Nat. Genet..

[bib31] Qian N. (1997). The IPL gene on chromosome 11p15.5 is imprinted in humans and mice and is similar to TDAG51, implicated in Fas expression and apoptosis. Hum. Mol. Genet..

[bib32] Rai A., Cross J.C. (2014). Development of the hemochorial maternal vascular spaces in the placenta through endothelial and vasculogenic mimicry. Dev. Biol..

[bib33] Salas M. (2004). Placental growth retardation due to loss of imprinting of Phlda2. Mech. Dev..

[bib34] Samaan N. (1968). Metabolic effects of placental lactogen (HPL) in man. J. Clin. Endocrinol. Metab..

[bib35] Schmittgen T.D., Livak K.J. (2008). Analyzing real-time PCR data by the comparative C(T) method. Nat. Protoc..

[bib36] Simmons D.G. (2008). Spatial and temporal expression of the 23 murine Prolactin/Placental Lactogen-related genes is not associated with their position in the locus. BMC Genom..

[bib37] Simmons D.G., Fortier A.L., Cross J.C. (2007). Diverse subtypes and developmental origins of trophoblast giant cells in the mouse placenta. Dev. Biol..

[bib38] Suzuki S. (2005). Genomic imprinting of IGF2, p57(KIP2) and PEG1/MEST in a marsupial, the tammar wallaby. Mech. Dev..

[bib39] Suzuki S. (2011). Characterisation of marsupial PHLDA2 reveals eutherian specific acquisition of imprinting. BMC Evol. Biol..

[bib40] Takao T. (2012). The maternally expressed gene Tssc3 regulates the expression of MASH2 transcription factor in mouse trophoblast stem cells through the AKT-Sp1 signaling pathway. J. Biol. Chem..

[bib41] Tunster S.J., Tycko B., John R.M. (2010). The imprinted Phlda2 gene regulates extraembryonic energy stores. Mol. Cell Biol..

[bib42] Tunster S.J., Van de Pette M., John R.M. (2011). Fetal overgrowth in the Cdkn1c mouse model of Beckwith-Wiedemann syndrome. Dis. Model. Mech..

[bib43] Tunster S.J., Van de Pette M., John R.M. (2012). Impact of genetic background on placental glycogen storage in mice. Placenta.

[bib44] Tunster S.J., Jensen A.B., John R.M. (2013). Imprinted genes in mouse placental development and the regulation of fetal energy stores. Reproduction.

[bib45] Tunster S.J., Van De Pette M., John R.M. (2014). Isolating the role of elevated Phlda2 in asymmetric late fetal growth restriction. Dis. Model. Mech..

[bib46] Withington S.L. (2006). Loss of Cited2 affects trophoblast formation and vascularization of the mouse placenta. Dev. Biol..

[bib47] Wu J.A. (2008). Murine pregnancy-specific glycoprotein 23 induces the proangiogenic factors transforming-growth factor beta 1 and vascular endothelial growth factor a in cell types involved in vascular remodeling in pregnancy. Biol. Reprod..

[bib48] Zhang Y., Szustakowski J., Schinke M. (2009). Bioinformatics analysis of microarray data. Methods Mol. Biol..

[bib49] Zheng-Fischhofer Q. (2007). Characterization of connexin31.1-deficient mice reveals impaired placental development. Dev. Biol..

