# Supplementary material for: The imprinted Phlda2 gene modulates a major endocrine compartment of the placenta to regulate placental demands for maternal resources
Source: Dev Biol. 2016 Jan 1;409(1):251–60. doi: 10.1016/j.ydbio.2015.10.015 (PMC4684229; doi:10.1016/j.ydbio.2015.10.015)
Supplement: Supplementary file 2 — Supplementary material [file mmc2.docx]

**Supplemental Table S1: QPCR data for Figures 1 and 2**

| **Figure 1** | |
| --- | --- |
| Gene | *Phlda2*^-/+^ (0X) |
| *Tpbpa* | 2.20 ± 0.26  *p* = 3.77 x 10^-4^ |
| *Prl8a8* | 2.10 ± 0.15  *p* = 8.78 x 10^-5^ |
| *Flt1* | 2.31 ± 0.51  *p* = 0.00551 |
| *Pcdh12* | 1.17 ± 0.12  *p* = 0.142 |
| *Gjb3* | 1.31 ± 0.33  *p* = 0.383 |
| *Flk1* | 0.93 ± 0.10  *p* = 0.506 |
| *Dlx3* | 0.71 ± 0.12  *p* = 0.0816 |
| *Syna* | 0.72 ± 0.09  *p* = 0.0542 |
| *Ly6e* | 0.75 ± 0.06  *p* = 0.0214 |
| *Gcm1* | 0.81 ± 0.09  *p* = 0.126 |
| *Cebpa* | 1.01 ± 0.15  *p* = 0.970 |
| *Tle3* | 0.92 ± 0.10  *p* = 0.489 |
| *Ctsq* | 0.86 ± 0.16  *p* = 0.446 |
| *Prl2c* | 1.38 ± 0.10  *p* = 0.00584 |
| *Prl3b1* | 1.54 ± 0.23  *p* = 0.0274 |

| **Figure 2** | | | |
| --- | --- | --- | --- |
| Gene | *Phlda2*^-/+^ (0X) | *Phlda2*^+/+BACx1^ (2X) | *Phlda2*^-/+BACx1^ (1X) |
| *Prl3c1* | 2.07 ± 0.45  *p* = 0.0162 | 0.48 ± 0.04  *p* = 7.50 x 10^-5^ | 0.72 ± 0.15  *p* = 0.202 |
| *Prl7a2* | 2.38 ± 0.45  *p* = 0.00472 | 0.51 ± 0.08  *p* = 0.0188 | 1.16 ± 0.19  *p* = 0.408 |
| *Prl8a1* | 2.79 ± 0.66  *p* = 0.0130 | 0.42 ± 0.09  *p* = 0.00355 | 0.89 ± 0.23  *p* = 0.667 |
| *Prl8a6* | 1.55 ± 0.35  *p* = 0.124 | 0.67 ± 0.08  *p* = 0.0164 | 0.79 ± 0.15  *p* = 0.251 |
| *Prl8a9* | 1.74 ± 0.29  *p* = 0.00983 | 0.48 ± 0.12  *p* = 0.0458 | 0.79 ± 0.18  *p* = 0.384 |
| *Psg17* | 1.89 ± 0.28  *p* = 0.00152 | 0.43 ± 0.09  *p* = 0.00678 | 0.92 ± 0.21  *p* = 0.711 |
| *Psg18* | 2.41 ± 0.65  *p* = 0.0139 | 0.38 ± 0.05  *p* = 0.00147 | 1.13 ± 0.25  *p* = 0.610 |
| *Psg19* | 3.31 ± 0.96  *p* = 0.0344 | 0.47 ± 0.10  *p* = 0.0180 | 1.08 ± 0.33  *p* = 0.817 |
| *Psg21* | 1.65 ± 0.29  *p* = 0.0138 | 0.42 ± 0.10  *p* = 0.0119 | 0.86 ± 0.17  *p* = 0.466 |

**Supplemental Table S2: KEGG pathways from unselected data set**

| **Term** | **Count** | **%** | **P Value** |
| --- | --- | --- | --- |
| mmu04110:Cell cycle | 17 | 1.644 | 1.2x10^-3^ |
| mmu04114:Oocyte meiosis | 15 | 1.451 | 3.1x10^-3^ |
| mmu03030:DNA replication | 7 | 0.677 | 9.7x10^-3^ |
| mmu04060:Cytokine-cytokine receptor interaction | 23 | 2.224 | 1.1x10^-2^ |
| mmu00600:Sphingolipid metabolism | 7 | 0.677 | 2.3x10^-2^ |
| mmu04914:Progesterone-mediated oocyte maturation | 10 | 0.967 | 3.7x10^-2^ |
| mmu04350:TGF-beta signaling pathway | 10 | 0.967 | 4.2x10^-2^ |
| mmu05219:Bladder cancer | 6 | 0.580 | 7.2x10^-2^ |
| mmu04115:p53 signaling pathway | 8 | 0.774 | 7.4x10^-2^ |
| mmu00590:Arachidonic acid metabolism | 9 | 0.870 | 7.5x10^-2^ |
| mmu00510:N-Glycan biosynthesis | 6 | 0.580 | 9.7x10^-2^ |

**Supplemental Table S4: KEGG pathways of UP DOWN data set**

| **Term** | **Count** | **%** | **P Value** |
| --- | --- | --- | --- |
| mmu04110:Cell cycle | 30 | 1.232 | 9x10^-4^ |
| mmu05210:Colorectal cancer | 22 | 0.903 | 2x10^-3^ |
| mmu00520:Amino sugar and nucleotide sugar metabolism | 14 | 0.575 | 2x10^-3^ |
| mmu05200:Pathways in cancer | 58 | 2.382 | 4x10^-3^ |
| mmu03010:Ribosome | 21 | 0.862 | 6x10^-3^ |
| mmu03030:DNA replication | 11 | 0.452 | 9x10^-3^ |
| mmu00510:N-Glycan biosynthesis | 13 | 0.534 | 9x10^-3^ |
| mmu04914:Progesterone-mediated oocyte maturation | 19 | 0.780 | 2x10^-2^ |
| mmu05216:Thyroid cancer | 9 | 0.370 | 2x10^-2^ |
| mmu04060:Cytokine-cytokine receptor interaction | 42 | 1.725 | 3x10^-2^ |
| mmu05212:Pancreatic cancer | 16 | 0.657 | 3x10^-2^ |
| mmu00052:Galactose metabolism | 8 | 0.329 | 4x10^-2^ |
| mmu04520:Adherens junction | 16 | 0.657 | 5x10^-2^ |
| mmu05222:Small cell lung cancer | 17 | 0.698 | 6x10^-2^ |
| mmu04810:Regulation of actin cytoskeleton | 36 | 1.478 | 6x10^-2^ |
| mmu00051:Fructose and mannose metabolism | 9 | 0.370 | 8x10^-2^ |
| mmu03040:Spliceosome | 22 | 0.903 | 9x10^-2^ |

**Supplemental Table S5: Functional annotation clustering of UP DOWN set**

| **Category** | **Term** | **Count** | **%** | **P Value** |
| --- | --- | --- | --- | --- |
| **Annotation Cluster 1** | **Enrichment Score: 7.178072621995058** |  |  |  |
| GOTERM_CC_FAT | GO:0043232~intracellular non-membrane-bounded organelle | 306 | 12.6 | 2.8x10^-9^ |
| GOTERM_CC_FAT | GO:0043228~non-membrane-bounded organelle | 306 | 12.6 | 2.8x10^-9^ |
| GOTERM_CC_FAT | GO:0005856~cytoskeleton | 191 | 7.8 | 5.5x10^-8^ |
| GOTERM_CC_FAT | GO:0044430~cytoskeletal part | 136 | 5.6 | 1.1x10^-6^ |
| SP_PIR_KEYWORDS | cytoskeleton | 108 | 4.4 | 2.7x10^-6^ |
| **Annotation Cluster 2** | **Enrichment Score: 5.701547121207655** |  |  |  |
| SP_PIR_KEYWORDS | mitosis | 50 | 2.05 | 1.3x10^-8^ |
| GOTERM_BP_FAT | GO:0000087~M phase of mitotic cell cycle | 50 | 2.05 | 2.3x10^-7^ |
| GOTERM_BP_FAT | GO:0007067~mitosis | 48 | 1.97 | 7.6x10^-7^ |
| GOTERM_BP_FAT | GO:0000280~nuclear division | 48 | 1.97 | 7.6x10^-7^ |
| GOTERM_BP_FAT | GO:0000278~mitotic cell cycle | 57 | 2.34 | 9.6x10^-7^ |
| GOTERM_BP_FAT | GO:0007049~cell cycle | 114 | 4.68 | 1.2x10^-6^ |
| GOTERM_BP_FAT | GO:0022402~cell cycle process | 80 | 3.29 | 2.0x10^-6^ |
| SP_PIR_KEYWORDS | cell cycle | 88 | 3.61 | 2.1x10^-6^ |
| GOTERM_BP_FAT | GO:0048285~organelle fission | 48 | 1.97 | 2.3x10^-6^ |
| SP_PIR_KEYWORDS | cell division | 56 | 2.30 | 8.8x10^-6^ |
| GOTERM_BP_FAT | GO:0000279~M phase | 60 | 2.46 | 1.3x10^-5^ |
| GOTERM_BP_FAT | GO:0022403~cell cycle phase | 67 | 2.75 | 1.3x10^-5^ |
| GOTERM_BP_FAT | GO:0051301~cell division | 55 | 2.26 | 2.7x10^-4^ |
| **Annotation Cluster 3** | **Enrichment Score: 4.253352104479643** |  |  |  |
| INTERPRO | IPR001400:Somatotropin hormone | 15 | 0.62 | 2.7x10^-7^ |
| INTERPRO | IPR018116:Somatotropin hormone, conserved site | 16 | 0.66 | 3.6x10^-7^ |
| PIR_SUPERFAMILY | PIRSF001825:prolactin/lactogen/growth hormone | 14 | 0.57 | 1.2x10^-6^ |
| INTERPRO | IPR012351:Four-helical cytokine, core | 18 | 0.74 | 2.2x10^-3^ |
| GOTERM_MF_FAT | GO:0005179~hormone activity | 24 | 0.99 | 8.9x10^-3^ |
| SP_PIR_KEYWORDS | hormone | 21 | 0.86 | 1.3x10^-2^ |
| **Annotation Cluster 4** | **Enrichment Score: 4.178045362781418** |  |  |  |
| GOTERM_MF_FAT | GO:0008092~cytoskeletal protein binding | 81 | 3.33 | 1.58x10^-5^ |
| SP_PIR_KEYWORDS | actin-binding | 50 | 2.05 | 2.05x10^-5^ |
| GOTERM_MF_FAT | GO:0003779~actin binding | 59 | 2.42 | 7.01x10^-5^ |
| GOTERM_CC_FAT | GO:0015629~actin cytoskeleton | 41 | 1.68 | 8.51x10^-4^ |
| **Annotation Cluster 5** | **Enrichment Score: 3.7304414445143914** |  |  |  |
| SP_PIR_KEYWORDS | ubl conjugation | 98 | 4.02 | 5.39x10^-6^ |
| SP_PIR_KEYWORDS | isopeptide bond | 54 | 2.22 | 3.09x10^-4^ |
| UP_SEQ_FEATURE | cross-link:Glycyl lysine isopeptide (Lys-Gly) (interchain with G-Cter in ubiquitin) | 34 | 1.40 | 0.00387 |
| **Annotation Cluster 6** | **Enrichment Score: 3.5295975689398236** |  |  |  |
| INTERPRO | IPR011993:Pleckstrin homology-type | 60 | 2.46 | 2.33x10^-5^ |
| INTERPRO | IPR001849:Pleckstrin homology | 54 | 2.22 | 6.86x10^-5^ |
| SMART | SM00233:PH | 54 | 2.22 | 3.71x10^-4^ |
| UP_SEQ_FEATURE | domain:PH | 37 | 1.52 | 0.0128 |
| **Annotation Cluster 7** | **Enrichment Score: 2.77467876913913** |  |  |  |
| GOTERM_CC_FAT | GO:0005694~chromosome | 67 | 2.75 | 6.3x10^-4^ |
| GOTERM_CC_FAT | GO:0000775~chromosome, centromeric region | 25 | 1.03 | 2.2x10^-3^ |
| GOTERM_CC_FAT | GO:0044427~chromosomal part | 55 | 2.26 | 3.5x10^-3^ |
| **Annotation Cluster 8** | **Enrichment Score: 2.747379236549496** |  |  |  |
| GOTERM_CC_FAT | GO:0015630~microtubule cytoskeleton | 88 | 3.61 | 1.7x10^-6^ |
| GOTERM_CC_FAT | GO:0005874~microtubule | 49 | 2.01 | 1.5x10^-4^ |
| GOTERM_BP_FAT | GO:0007017~microtubule-based process | 45 | 1.85 | 1.5x10^-4^ |
| UP_SEQ_FEATURE | domain:Kinesin-motor | 13 | 0.53 | 6.1x10^-4^ |
| SP_PIR_KEYWORDS | microtubule | 42 | 1.72 | 2.5x10^-3^ |
| INTERPRO | IPR019821:Kinesin, motor region, conserved site | 13 | 0.53 | 2.9x10^-3^ |
| INTERPRO | IPR001752:Kinesin, motor region | 13 | 0.53 | 4.4x10^-3^ |
| GOTERM_MF_FAT | GO:0003777~microtubule motor activity | 18 | 0.74 | 5.9x10^-3^ |
| SMART | SM00129:KISc | 13 | 0.53 | 7.7x10^-3^ |
| GOTERM_BP_FAT | GO:0007018~microtubule-based movement | 22 | 0.90 | 7.8x10^-3^ |
| SP_PIR_KEYWORDS | motor protein | 22 | 0.90 | 5.6x10^-2^ |
| GOTERM_MF_FAT | GO:0003774~motor activity | 24 | 0.99 | 7.3x10^-2^ |
| **Annotation Cluster 9** | **Enrichment Score: 2.6021182424648677** |  |  |  |
| GOTERM_BP_FAT | GO:0007010~cytoskeleton organization | 65 | 2.67 | 4x10^-5^ |
| GOTERM_BP_FAT | GO:0030036~actin cytoskeleton organization | 32 | 1.31 | 7x10^-3^ |
| GOTERM_BP_FAT | GO:0030029~actin filament-based process | 33 | 1.36 | 1x10^-2^ |
| GOTERM_BP_FAT | GO:0007015~actin filament organization | 14 | 0.57 | 1x10^-2^ |
| **Annotation Cluster 10** | **Enrichment Score: 2.445796361217853** |  |  |  |
| GOTERM_MF_FAT | GO:0060589~nucleoside-triphosphatase regulator activity | 74 | 3.04 | 1.3x10^-5^ |
| GOTERM_MF_FAT | GO:0030695~GTPase regulator activity | 73 | 3.00 | 1.4x10^-5^ |
| GOTERM_MF_FAT | GO:0008047~enzyme activator activity | 53 | 2.18 | 6.1x10^-5^ |
| GOTERM_MF_FAT | GO:0005096~GTPase activator activity | 42 | 1.72 | 2.2x10^-4^ |
| GOTERM_BP_FAT | GO:0051056~regulation of small GTPase mediated signal transduction | 47 | 1.93 | 2.5x10^-4^ |
| GOTERM_MF_FAT | GO:0005083~small GTPase regulator activity | 45 | 1.85 | 7.8x10^-4^ |
| SP_PIR_KEYWORDS | guanine-nucleotide releasing factor | 23 | 0.94 | 3.9x10^-3^ |
| GOTERM_BP_FAT | GO:0046578~regulation of Ras protein signal transduction | 35 | 1.44 | 5.0x10^-3^ |
| SP_PIR_KEYWORDS | GTPase activation | 28 | 1.15 | 5.1x10^-3^ |
| GOTERM_MF_FAT | GO:0005085~guanyl-nucleotide exchange factor activity | 29 | 1.19 | 1.1x10^-2^ |
| GOTERM_BP_FAT | GO:0035023~regulation of Rho protein signal transduction | 19 | 0.78 | 1.2x10^-2^ |
| INTERPRO | IPR000219:Dbl homology (DH) domain | 15 | 0.62 | 2.0x10^-2^ |
| GOTERM_MF_FAT | GO:0005089~Rho guanyl-nucleotide exchange factor activity | 16 | 0.66 | 2.2x10^-2^ |
| SMART | SM00325:RhoGEF | 15 | 0.62 | 3.3x10^-2^ |
| UP_SEQ_FEATURE | domain:PH 2 | 9 | 0.37 | 3.5x10^-2^ |
| UP_SEQ_FEATURE | domain:DH | 13 | 0.53 | 3.6x10^-2^ |
| UP_SEQ_FEATURE | domain:PH 1 | 9 | 0.37 | 4.2x10^-2^ |
| GOTERM_MF_FAT | GO:0005088~Ras guanyl-nucleotide exchange factor activity | 16 | 0.66 | 7.5x10^-2^ |
| INTERPRO | IPR001331:Guanine-nucleotide dissociation stimulator, CDC24, conserved site | 11 | 0.45 | 9.4x10^-2^ |

**Supplemental Table S6: UP DOWN set Cluster 3 Gene report of significant changes in gene expression**

| **Cluster 3** | ***Phlda2*^-/+^** | | ***Phlda2*^+/+BACx1^** | |
| --- | --- | --- | --- | --- |
| Gene Name | Fold up | P Value | Fold down | P value |
| prolactin family 2, subfamily c, member 1 | 1.36 | 7.1x10^-4^ | 1.91 | 9.2x10^-7^ |
| prolactin family 2, subfamily a, member 1 | 1.13 | 1.9x10^-2^ | 1.16 | 1.9x10^-2^ |
| prolactin family 3, subfamily c, member 1 | 1.20 | 1.3x10^-2^ | 1.96 | 9.1x10^-3^ |
| prolactin family 5, subfamily a, member 1 | 1.56 | 1.5x10^-2^ | 2.82 | 9.7x10^-5^ |
| prolactin family 7, subfamily b, member 1 | 1.32 | 2.0x10^-3^ | 1.53 | 2.3x10^-3^ |
| prolactin family 8, subfamily a, member 6 | 1.10 | 2.9x10^-2^ | 1.69 | 2.6x10^-3^ |
| inhibin beta-B | 1.17 | 3.9x10^-2^ | 1.48 | 2.4x10^-4^ |
| interleukin 3 | 1.16 | 7.4x10^-3^ | 1.26 | 2.2x10^-3^ |
| oncostatin M | 1.10 | 4.8x10^-2^ | 1.10 | 4.8x10^-2^ |
| secretin | 1.17 | 2.4x10^-2^ | 1.35 | 4.3x10^-4^ |

**Supplemental Table S7: Data for Figure 4 (4-7 litters for each time point)**

| A: Fetal weight | | | |
| --- | --- | --- | --- |
|  | E14.5 | E16.5 | E18.5 |
| *Phlda2*^+/+^ | 216.4 mg ± 4.5  n = 15 | 646.3 mg ± 19.1  n = 14 | 1038.1 mg ± 22.5  n = 20 |
| *Phlda2*^-/+^ | 215.2 mg ± 3.0  n = 14 | 680.4 mg ± 13.7  n = 14 | 1065.0 mg ± 19.0  n = 24 |
| Ratio and *p* value  (t-test) | 99.5%  *p* = 0.835 | 105.3%  *p* = 0.159 | 102.6%  *p* = 0.361 |
| B: Placental weight | | | |
| *Phlda2*^+/+^ | 66.5 mg ± 2.0  n = 15 | 73.4 mg ± 1.3  n = 14 | 72.8 mg ± 0.9  n = 20 |
| *Phlda2*^-/+^ | 80.4 mg ± 2.0  n = 14 | 107.9 mg ± 4.5  n = 14 | 93.6 mg ± 2.7  n = 24 |
| Ratio and *p* value  (t-test) | 121.0%  *p* = 3.21 x 10^-5^ | 146.9%  *p* = 7.42 x 10^-8^ | 128.7%  *p* = 3.10 x 10^-8^ |
| C: F:P Ratio | | | |
| *Phlda2*^+/+^ | 3.3 ± 0.11  n = 15 | 8.8 ± 0.26  n = 14 | 14.3 ± 0.33  n = 20 |
| *Phlda2*^-/+^ | 2.7 ± 0.06  n = 14 | 6.4 ± 0.30  n = 14 | 11.6 ± 0.36  n = 24 |
| Ratio and *p* value  (t-test) | 81.7%  *p* = 7.39 x 10^-5^ | 73.1%  *p* = 2.75 x 10^-6^ | 81.0%  *p* = 2.10 x 10^-6^ |
| D: Glycogen (mg) | | | |
|  | E14.5 | E16.5 | E18.5 |
| *Phlda2*^+/+^ | 0.282 mg ± 0.047  n = 13 | 0.208 mg ± 0.036  n = 13 | 0.255 mg ± 0.031  n = 20 |
| *Phlda2*^-/+^ | 0.336 mg ± 0.037  n = 12 | 0.488 mg ± 0.041  n = 17 | 0.427 mg ± 0.031  n = 24 |
| Ratio and *p* value  (t-test) | 119.1%  *p* = 0.381 | 234.4%  *p* = 3.01 x 10^-5^ | 167.5%  *p* = 3.55 x 10^-4^ |
| E: Glycogen (mg/g) (129) | | | |
| *Phlda2*^+/+^ | 4.23 mg/g ± 0.67  n = 13 | 2.82 mg/g ± 0.35  n = 13 | 3.46 mg/g ± 0.37  n = 20 |
| *Phlda2*^-/+^ | 4.26 mg/g ± 0.49  n = 12 | 4.77 mg/g ± 0.41  n = 17 | 4.52 mg/g ± 0.29  n = 24 |
| Ratio and *p* value  (t-test) | 100.5%  *p* = 0.979 | 169.3%  *p* = 1.08 x 10^-3^ | 130.6%  *p* = 0.0292 |

**Supplemental Table S8: Data for Figure 5 (6-8 litters for each strain)**

| A: Fetal weight (E18.5) | | | | | |
| --- | --- | --- | --- | --- | --- |
|  | | 129 | | BL6 | |
| *Phlda2*^+/+^ | | 1228.9 mg ± 10.5  n = 39 | | 1175.0 mg ± 13.2  n = 41 | |
| *Phlda2*^+/+^ (non transgenic littermates) | | 1038.1 mg ± 22.5  n = 20 | | 1133.2 mg ± 14.1  n = 33 | |
| *Phlda2*^-/+^ | | 1065.0 mg ± 19.0  n = 24 | | 1123.3 mg ± 29.6  n = 24 | |
| ANOVA | | *F*_2,80_ = 46.9, *p* = 3.28 x 10^-14^ | | *F*_2,95_ = 2.51, *p* = 0.0865 | |
| Ratio and Bonferroni corrected *p* value *Phlda2*^+/+^ vs *Phlda2*^+/+WT^ | | 84.5%  *p* = 5.16 x 10^-12^ | | 96.4%  *p* = 0.245 | |
| Ratio and Bonferroni corrected *p* value *Phlda2*^-/+^ vs *Phlda2*^+/+WT^ | | 86.7%  *p* = 1.50 x 10^-10^ | | 95.6%  *p* = 0.151 | |
| Ratio and Bonferroni corrected *p* value *Phlda2*^-/+^ vs *Phlda2*^+/+^ | | 102.6%  *p* = 0.863 | | 99.1%  *p* = 1.000 | |
| B: Placental weight (E18.5) | | | | | |
| *Phlda2*^+/+^ | | 77.6 mg ± 1.1  n = 39 | | 98.5 mg ± 1.5  n = 41 | |
| *Phlda2*^+/+^(non transgenic littermates) | | 72.8 mg ± 0.9  n = 20 | | 92.1 mg ± 1.3  n = 33 | |
| *Phlda2*^-/+^ | | 93.6 mg ± 2.7  n = 24 | | 112.6 mg ± 3.6  n = 24 | |
| ANOVA | | *F*_2,80_ = 36.0, *p* = 6.93 x 10^-12^ | | *F*_2,95_ = 22.7, *p* = 9.02 x 10^-9^ | |
| Ratio and Bonferroni corrected *p* value *Phlda2*^+/+^ vs *Phlda2*^+/+WT^ | | 93.8%  *p* = 0.152 | | 93.5%  *p* = 0.0580 | |
| Ratio and Bonferroni corrected *p* value *Phlda2*^-/+^ vs *Phlda2*^+/+WT^ | | 120.7%  *p* = 2.23 x 10^-9^ | | 114.3%  *p* = 1.84 x 10^-5^ | |
| Ratio and Bonferroni corrected *p* value *Phlda2*^-/+^ vs *Phlda2*^+/+^ | | 128.7%  *p* = 6.19 x 10^-11^ | | 122.2%  *p* = 5.22 x 10^-9^ | |
| C: F:P Ratio (E18.5) | | | | | |
| *Phlda2*^+/+^ | | 15.9 ± 0.22  n = 39 | | 12.0 ± 0.16  n = 41 | |
| *Phlda2*^+/+^(non transgenic littermates) | | 14.3 ± 0.33  n = 20 | | 12.4 ± 0.18  n = 33 | |
| *Phlda2*^-/+^ | | 11.6 ± 0.36  n = 24 | | 10.1 ± 0.26  n = 24 | |
| ANOVA | | *F*_2,80_ = 61.7, *p* = 6.25 x 10^-17^ | | *F*_2,95_ = 33.2, *p* = 1.15 x 10^-11^ | |
| Ratio and Bonferroni corrected *p* value *Phlda2*^+/+^ vs *Phlda2*^+/+WT^ | | 89.7%  *p* = 5.87 x 10^-4^ | | 103.0%  *p* = 0.521 | |
| Ratio and Bonferroni corrected *p* value *Phlda2*^-/+^ vs *Phlda2*^+/+WT^ | | 72.6%  *p* = 2.62 x 10^-17^ | | 84.1%  *p* = 2.16 x 10^-9^ | |
| Ratio and Bonferroni corrected *p* value *Phlda2*^-/+^ vs *Phlda2*^+/+^ | | 81.0%  *p* = 2.22 x 10^-7^ | | 81.7%  *p* = 2.88 x 10^-11^ | |
| D: E18.5 Glycogen (mg) and E: Glycogen (mg/g) compared with true 129 WT | | | | |  |
| *Phlda2*^+/+^ | 0.181 mg ± 0.013  n = 24 | | 2.36 mg/g ± 0.13  n = 24 | |  |
| *Phlda2*^+/+^(non transgenic littermates) | 0.255 mg ± 0.031  n = 20 | | 3.46 mg/g ± 0.37  n = 20 | |  |
| *Phlda2*^-/+^ | 0.427 mg ± 0.031  n = 24 | | 4.52 mg/g ± 0.29  n = 24 | |  |
| ANOVA | *F*_2,65_ = 24.9, *p* = 9.47 x 10^-9^ | | *F*_2,65_ = 16.5, *p* = 1.65 x 10^-6^ | |  |
| Ratio and Bonferroni corrected *p* value *Phlda2*^+/+^ vs *Phlda2*^+/+WT^ | 140.9%  *p* = 0.153 | | 146.5%  *p* = 0.0210 | |  |
| Ratio and Bonferroni corrected *p* value Phlda2^-/+^ vs *Phlda2*^+/+WT^ | 235.9%  *p* = 7.62 x 10^-9^ | | 191.3%  *p* = 8.10 x 10^-7^ | |  |
| Ratio and Bonferroni corrected *p* value Phlda2^-/+^ vs *Phlda2*^+/+^ | 167.5%  *p* = 6.09 x 10^-5^ | | 130.6%  *p* = 0.0274 | |  |

**Supplemental Table S9: Data for Figure 6 (wild type model N = 5 and *Phlda2*^-/+^ model N = 8 litters)**

| E18.5 Embryo Transfer | | | | |
| --- | --- | --- | --- | --- |
|  | A: Fetal weight | B: Placenta weight | | C: F:P Ratio |
| *Phlda2*^+/+WT^ | 1152.2 mg ± 16.3  n = 35 | 71.6 mg ± 1.4  n = 35 | | 16.3 ± 0.3  n = 35 |
| *Phlda2*^+/+^ (non transgenic littermates) | 1062.6 mg ± 10.6  n = 30 | 69.9 mg ± 1.8  n = 30 | | 15.5 ± 0.4  n = 30 |
| *Phlda2*^-/+^ | 1014.7 mg ± 25.3  n = 24 | 79.0 mg ± 3.4  n = 24 | | 13.2 ± 0.5  n = 24 |
| ANOVA | *F*_2,86_ = 16.4,  *p* = 9.56 x 10^-7^ | *F*_2,86_ = 4.51,  *p* = 0.0137 | | *F*_2,86_ = 15.0,  *p* = 2.50 x 10^-6^ |
| Ratio and Bonferroni corrected *p* value *Phlda2*^+/+^ vs *Phlda2*^+/+WT^ | 92.2%  *p* = 7.74 x 10^-4^ | 97.6%  *p* = 1.000 | | 95.0%  *p* = 0.379 |
| Ratio and Bonferroni corrected *p* value Phlda2^-/+^ vs *Phlda2*^+/+WT^ | 87.9%  *p* = 1.18 x 10^-6^ | 112.8%  *p* = 0.0552 | | 81.2%  *p* = 1.61 x 10^-6^ |
| Ratio and Bonferroni corrected *p* value Phlda2^-/+^ vs *Phlda2*^+/+^ | 95.5%  *p* = 0.202 | 113.1%  *p* = 0.0161 | | 85.5%  *p* = 6.98 x 10^-4^ |
|  | D: Glycogen (mg) | | E: Glycogen (mg/g) | |
| *Phlda2*^+/+WT^ | 0.082 mg ± 0.010  n = 35 | | 1.23 mg/g ± 0.12  n = 35 | |
| *Phlda2*^+/+^ (non transgenic littermates) | 0.140 mg ± 0.013  n = 30 | | 1.98 mg/g ± 0.16  n = 30 | |
| *Phlda2*^-/+^ | 0.253 mg ± 0.017  n = 24 | | 3.11 mg/g ± 0.19  n = 24 | |
| ANOVA | *F*_2,86_ = 41.5, *p* = 2.39 x 10^-13^ | | *F*_2,86_ = 35.8, *p* = 2.39 x 10^-13^ | |
| Ratio and Bonferroni corrected *p* value *Phlda2*^+/+^ vs *Phlda2*^+/+WT^ | 170.8%  *p* = 0.00467 | | 161.4%  *p* = 0.00154 | |
| Ratio and Bonferroni corrected *p* value Phlda2^-/+^ vs *Phlda2*^+/+WT^ | 309.2%  *p* = 8.00 x 10^-14^ | | 242.4%  *p* = 1.77 x 10^-12^ | |
| Ratio and Bonferroni corrected *p* value Phlda2^-/+^ vs *Phlda2*^+/+^ | 309.2%  *p* = 2.42 x 10^-7^ | | 157.0%  *p* = 1.26 x 10^-5^ | |
